# Research on Area of Uncertainty of Underwater Moving Target Based on Stochastic Maneuvering Motion Model

**DOI:** 10.3390/s22228837

**Published:** 2022-11-15

**Authors:** Shasha Ma, Haiyan Wang, Xiaohong Shen, Zhenxin Sun, Ning Sun

**Affiliations:** 1 School of Marine Science and Technology, Northwestern Polytechnical University, Xi’an 710072, China; 2 Jiangsu Automation Research Institute, Lianyungang 222061, China

**Keywords:** area of uncertainty, underwater target tracking, Kalman filter, stochastic maneuvering motion model

## Abstract

Considering the influence of measurement error on target state estimation, there is an uncertain dispersion region for target position estimate, that is, the area of uncertainty (AOU, area of uncertainty). In underwater target tracking, the state estimation is point estimation without AOU estimation and its accuracy is poor in the early stage because of large measurement errors. Fast tracking with higher accuracy and AOU estimation are of great significance to time-sensitive target tracking. To improve the state estimation accuracy in the early stage, and estimate the AOU, a method of AOU estimation of underwater moving target is presented based on a stochastic maneuvering motion (SMM, stochastic maneuvering motion) model. The stochastic maneuvering motion model is established based on the Langevin equation to reflect the movement characteristics of an underwater moving target. Then, the target state is estimated with a noise adaptive Kalman filter by constructing the measurement equation and state equation according to measurement error characteristic and stochastic maneuvering model. Based on the physical significance of the error covariance matrix from the Kalman filter, the parameters of AOU are deduced. Simulation results of underwater target tracking and AOU estimation are presented to demonstrate the relative performance of the proposed algorithm compared with the adaptive Kalman filter. It is clearly shown from the results that SMM tracking algorithm achieves higher accuracy of state estimation in the initial stage of tracking, and the predicted AOU is consistent with the actual distribution of underwater moving targets while yielding more concentrated distribution, which reveals that estimated AOU can be precisely represented by the confidence ellipses. The presented approach and obtained results may be useful in time-sensitive target threat analysis and weapon strike applications.

## 1. Introduction

Target tracking is a very important technology since it plays an important role in many applications. Real-time estimation is always a crucial consideration for tracking algorithms, especially for time sensitive target tracking. Range and bearing measurements of sensors are widely used in target tracking. However, the state estimation results are point estimation and yield appropriate estimators of the target position. Because of the measurement error of the sensor, especially the large measurement error of the remote target, the estimated state, as well as the predicted value of the target state, changes greatly; therefore, the point estimation cannot effectively reflect the error level of the estimated target state. Considering the influence of measurement error on the estimation error of target state, there is an uncertain dispersion region under a confidence degree, that is, the area of uncertainty (AOU) of the target. With the search ability improvement of intelligent weapons, the attack mode is no longer concerned with hitting a single point, but to make the searching area of the weapon consistent with the area of uncertainty of a moving target, which aims to maximize the weapon’ s capture probability. Therefore, accurate estimation of the AOU is of great importance, as well as the target motion parameters for the precision strike of intelligent weapons.

In recent years, researchers have suggested many efficient algorithms for target state estimation in the field of underwater target tracking [1,2,3,4,5,6,7,8,9]. In the existing literature, underwater target tracking algorithms are usually based on a Kalman filter, extended Kalman filter (EKF), unscented Kalman filter (UKF), and particle Filter (PF). As bearing measurements are nonlinear to the target state, EKF [10,11] and PF are adopted in many bearing-only tracking cases [12,13,14,15,16,17]. However, EKF has limitations, such as track divergence and poor estimation accuracy, particularly for high initial errors [18]. To improve the estimation accuracy, a few deterministic sample point filters, such as the unscented Kalman filter (UKF) [19,20], Gauss-Hermite filter (GHF) [21,22], and cubature Kalman filter (CKF) [23,24], have been studied. In these filters, probability density functions are required and may result in error covariance to be asymmetric and non-positive definite [25]. Because the angle changes little during the sampling interval for the limited speed of the target compared with the long distance between targets and receivers, underwater target maneuvering can be described as an approximate linear model in practice [26]. Based on this concept, the authors in [27] used KF, which is an optimal estimation algorithm with lower computation complexity compared with EKF, UKF, and PF, to track the underwater target. In [28], an adaptive KF was proposed by estimating the process noise variance, which effectively improved the tracking accuracy of an underwater maneuvering target.

As most the target tracking problems have been investigated based on a target motion model, this may lead to the divergence of the subsequent filtering process if the model is far different from the actual situation [29]. In the marine environment, there are three frequently used target motion models: the constant velocity (CV) model, the constant acceleration (CA) model, and the turning model [26]. The tracking performances of EKF, UKF, and PF for an underwater target with different motion models such as CA, CV, and the variable speed linear model were investigated in [30], the results indicating that all EKF, UKF, PF based methods can provide accurate tracking results for the linear model, while PF has superior performance when systems are nonlinear and with non-Gaussian noise. However, the measurement error is large due to the complexity of the underwater environment, and may result in large tracking error even employing filtering techniques, especially at the initial stage of target tracking. For a time-sensitive target, it is essential to improve the tracking performance as quickly as possible while the existing methods do not achieve fast tracking under the condition of a large measurement error.

For AOU estimation, the uncertainty region of the stationary and passive target has been investigated by researchers [31,32,33]. Precise expressions concerning two-dimensional confidence regions for an unknown position of object was presented in [31], and uncertainty regions were defined as error ellipses and confidence ellipses. The subject of estimating the position region of a passive stationary object was studied in [32,33]. On the other hand, the AOU of an active target, especially an underwater moving target, has received less attention. An example of such an estimation was investigated in [34], where the current AOU of a target moving in a straight line with constant velocity was estimated based on least square algorithm that recalculated all the measured information as the observation was updated; the algorithm was not applicable to AOU prediction. From a literature study, it is understood that the AOU prediction of active target motion analysis has not yet been studied; in our research work this topic is discussed in detail.

In this paper, an algorithm of AOU estimation based on a stochastic maneuvering motion model for an underwater target is proposed, where the accuracy of the predicted position is improved at the initial tracking stage, and the position uncertainty area is more concentrated. Our contributions can be summarized as follows. First, a stochastic maneuvering motion model for an underwater target is established based on the Langevin equation. Second, the underwater target state is estimated based on a stochastic maneuvering motion model with an adaptive noise Kalman filter. In addition, the parameters of the area of uncertainty are deduced based on the physical significance of the error covariance matrix.

This article is organized as follows. In Section 2, we describe the problem of AOU and discuss the influencing factors. Section 3 formulates and presents an underwater target tracking algorithm based on stochastic maneuvering motion model. In Section 4, the parameters of AOU are deduced based on the physical significance of the error covariance matrix. Comprehensive simulation studies are presented in Section 5. The article ends in Section 6 with concluding remarks.

## 2. Problem Statement

**Figure 1:**
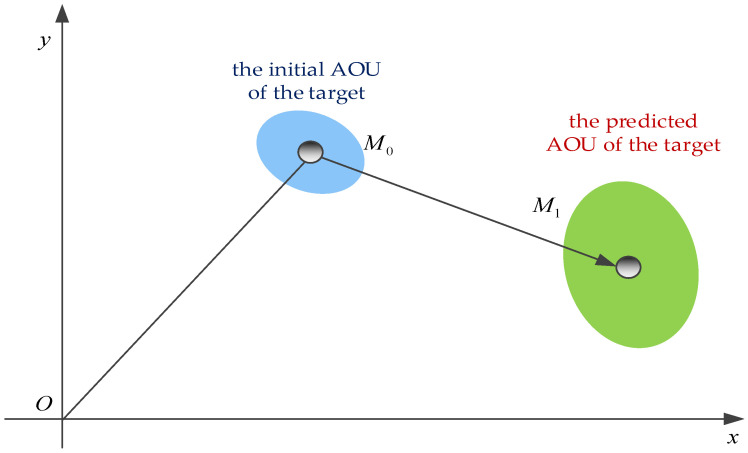
The schematic diagram of AOU for moving target.

The size of AOU of the moving target is affected by two factors, the measurement error and the state estimation error. A schematic diagram of AOU of a moving target is shown in Figure 1. Due to the limitation of sensor performance, the measurements inevitably have error, which leads to error in the initial position of the target. The initial AOU of the target is the ellipse region centered on the initial detected position $M_{0}$. On the other hand, it takes a long time for a long-range attack weapon to reach the designated target, during which the target is still moving, and the error of target state estimation leads to the significant expansion of the predicted target position error. Thus, the predicted AOU of the target is the ellipse region centered on the predicted position $M_{1}$. Therefore, to estimate AOU of the moving target accurately, the target state estimation should be calculated effectively as well as the error characteristics of state estimation.

## 3. State Estimation of an Underwater Moving Target

To estimate the state of an underwater moving target, a stochastic maneuvering motion model was adopted which conforms to the motion characteristics of underwater target. Then the target measurement equation and state equation are established in Cartesian coordinate system. The target motion state is estimated accurately by using noise-adaptive Kalman filter [26], and finally the AOU of underwater target is calculated according to the predicted position and covariance.

### 3.1. Stochastic Maneuvering Motion Model

When the target moves underwater, it is affected by a variety of marine physical factors. The external forces the underwater target receives are mainly divided into two categories. One has a macroscopic and continuous effect, and can be represented as viscous resistance in Stokes’ theorem. The other type has only microscopic effects and is changing constantly, mathematically represented by the Wiener process. The kinematic equation of the underwater target was established by synthesizing the above two forces; that is the Langevin Equation [34] as shown in Equation (2).

Let X(t) denotes the target position in Cartesian coordinate system and V(t) denotes the target velocity. V(t) is the derivative of X(t), that is

(1) $$V(t)=\frac{dX(t)}{dt}$$

The Langevin Equation, which reflects the underwater target state is a first-order linear stochastic differential equation as follows

(2) dV(t)=−βV(t)dt+σdW(t)

where −βV(t)dt represents the viscous resistance proportional to the target velocity, β is damping coefficient, σdW(t) denotes the random force of a large number of molecules on the target while W(t) is the Wiener process.

Let

$$\eta (t)=V(t)e^{\beta t}$$

Through $It\hat{o}$ Integration by the Parts Formula [35], the differentiation of η(t) can be written as follows:

(3) $$d\eta (t)=e^{\beta t}dV(t)+V(t)d(e^{\beta t})+d{\langle V,e^{\beta t}\rangle }_{t}$$

As $d(e^{\beta t})=\beta e^{\beta t}dV(t)+0dW(t)$,

Thus $d{\langle V,e^{\beta t}\rangle }_{t}=0$.

Equation (3) can be obtained from Equation (2) as the follow expression

(4) $$d\eta (t)=e^{\beta t}[-\beta V(t)dt+\sigma dW(t)]+\beta V(t)e^{\beta t}d(t)=\sigma e^{\beta t}dW(t)$$

That is

(5) $$\eta (t)=\eta (0)+\sigma \int_{0}^{t}e^{\beta s}dW(s)=V(0)+\sigma \int_{0}^{t}e^{\beta s}dW(s)$$

The ergodic solution of the stochastic differential Equation is obtained. The velocity of the target is shown in Equation (6)

(6) $$V(t)=\eta (t)e^{-\beta t}=V(0)e^{-\beta t}+\sigma e^{-\beta t}\int_{0}^{t}e^{\beta s}dW(s),\forall t\geq 0$$

Equation (6) describes the law of the target motion velocity and provides a suitable motion model for the establishment of the underwater target state equation.

### 3.2. Target State Equation 

Let X(k) denote the target state at time step k, that is

$$X(k)=\left[\begin{array}{c} x_{k}\\ v_{xk} \end{array}\right]$$

where $x_{k}$ is the position coordinates along x axis, and $v_{xk}$ is the velocity vector. Thus, based on Equation (2), the state equation can be described as

(7) $$\dot{X}(k)=\left[\begin{array}{cc} 0 & 1\\ 0 & -\beta \end{array}\right]\left[\begin{array}{c} x_{k}\\ v_{xk} \end{array}\right]+\left[\begin{array}{c} 0\\ \sigma \end{array}\right]dW(k)$$

Equation (7) can be abbreviated as follows

(8) $$\dot{X}(k)=F(k)X(k)+Gw(k)$$

where $F(k)=\left[\begin{array}{cc} 0 & 1\\ 0 & -\beta \end{array}\right]$ and $G=\left[\begin{array}{c} 0\\ \sigma \end{array}\right]$.

Let X(0) denote the initial state at time $t_{0}$. The target state at any time $t_{k}$($t_{k}>t_{0}$) can be obtained from Equation (6) as follows.

(9) $$X(k)=X(0)\Phi (t_{k},t_{0})+\int_{\tau =t_{0}}^{t_{k}}\Phi (t_{k},\tau )GdW(\tau )$$

where $\Phi (t_{k},t_{0})$ is the state transition matrix from $t_{0}$ to $t_{k}$. $\Phi (t_{k},t_{0})$ is given by

(10) $$\Phi (t_{k},t_{0})=e^{F(t_{k})(t_{k}-t_{0})}$$

As F(k) is time invariant, $\Phi (t_{k},t_{0})$ has nothing to do with initial time $t_{0}$. The state equation in the form of a differential equation can be obtained from the state transition matrix $\Phi (t_{k},t_{0})$ thus:

(11) $$\{\begin{array}{c} v(t_{k+1})=v(t_{k})e^{-\beta t}\\ x(t_{k+1})=x(t_{k})+\frac{1}{\beta }(v(t_{k})-v(t_{k+1})) \end{array}$$

As shown in Equation (11), the mean of the target velocity decays by $e^{-\beta t}$ times the previous velocity, and the change in target position is equal to $\beta^{-1}$ times the change in velocity. $\Phi (t_{k},t_{0})$ depends on the parameters β and $t_{k}$ only and has nothing to do with σ; thus, $\Phi (t_{k},t_{0})$ can be written as Φ(k). The system noise matrix Q is the noise variance matrix reflecting the Wiener process W(k) and can be reflected as follows:

(12) $$Q=E\left[\left(\int_{\tau =t_{0}}^{t_{k}}\Phi (t_{k},\tau )Gw(\tau )d\tau \right){\left(\int_{s=t_{0}}^{t_{k}}\Phi (t_{k},s)Gw(s)ds\right)}^{T}\right]=\int_{\tau =t_{0}}^{t_{k}}\Phi (t_{k},\tau )GG^{T}\Phi^{T}(t_{k},\tau )d\tau$$

Take Equation (10) into Equation (12), Q can be written as:

(13) $$Q=\int_{\tau =t_{0}}^{t_{k}}\Phi (t_{k},\tau )\left[\begin{array}{cc} 0 & 0\\ 0 & \sigma^{2} \end{array}\right]\Phi^{T}(t_{k},\tau )d\tau$$

Therefore, the system noise matrix Q is defined by β, σ and $t_{k}$.

### 3.3. Target Measurements Model

In target tracking, sensors generally provide range measurements r(k) and bearing measurements b(k) with error. In sensor coordinates, these measurements are expressed in the following form with additive noise:

$$\begin{array}{l} \bar{r}\left(k\right)=r\left(k\right)+v_{rk}\\ \bar{b}\left(k\right)=b\left(k\right)+v_{bk} \end{array}$$

where (r(k),b(k)) is the true value of target position in sensor polar coordinates at time $t_{k}$. $v_{rk}$ and $v_{bk}$ are random error of range and bearing measurements, which are random variables that obey zero-mean Gaussian distribution and uncorrelated with each other.

Let $t_{0}$ denote the initial time, $V_{W}$ and $C_{W}$ are velocity and course of the sensor platform, respectively. At time $t_{k}$, the range and bearing measurements of the underwater target are $\bar{r}\left(k\right)$ and $\bar{b}\left(k\right)$. Then at time $t_{k}$, the measured position coordinates of target in Cartesian coordinates are $\left({\bar{x}}_{k},{\bar{y}}_{k}\right)$ and can be described as:

$${\bar{x}}_{k}=\bar{r}\left(k\right)\sin\bar{b}\left(k\right)+V_{W}\cdot \sin C_{W}\cdot \left(t_{k}-t_{0}\right)$$

$${\bar{y}}_{k}=\bar{r}\left(k\right)\cos\bar{b}\left(k\right)+V_{W}\cdot \cos C_{W}\cdot \left(t_{k}-t_{0}\right)$$

Let $V_{k}$ denote noise covariance matrix. $V_{k}$ is a random vector with zero mean and it is independent of system noise matrix $W_{k}$. The measurement matrix Z(k) can be modeled as

$$Z\left(k\right)=\left[\begin{array}{c} {\bar{x}}_{k}\\ {\bar{y}}_{k} \end{array}\right]=HX\left(k\right)+V_{k}$$

The observation matrix H is

(14) $$H=\left[\begin{array}{c} \begin{array}{cccc} 1 & 0 & 0 & 0 \end{array}\\ \begin{array}{cccc} 0 & 1 & 0 & 0 \end{array} \end{array}\right]$$

### 3.4. Filtering Algorithm

It is assumed that at time $t_{k-1}$ the estimated state is, $\hat{X}(k-1|k-1)$ while the covariance matrix is. Then, the predicted state is

(15) $$\hat{X}(k|k-1)=\Phi (k-1)\hat{X}(k-1|k-1)$$

The predicted state covariance is

(16) $$P(k|k-1)=\Phi (k-1)P(k-1|k-1)\Phi^{T}(k-1)+Q$$

The predicted measurement matrix is

(17) $$\hat{Z}(k|k-1)=H(k)\hat{X}(k|k-1)$$

Let $\tilde{M}(k)$ denote the innovation vector:

(18) $$\tilde{M}(k)=Z(k)-H\hat{X}(k|k-1)=H\left[X(k)-\hat{X}(k|k-1)\right]+V(k)$$

$\tilde{M}(k)$ is a zero-mean white noise sequence. Take the variance of Equation (18) to obtain the variance matrix of innovation S(k):

$$S(k)=Var\left[\tilde{M}(k)\right]=HP(k|k-1)H^{T}+R(k)$$

Then the estimation of R(k) is

(19) $$\hat{R}(k)=\hat{S}(k)-HP(k|k-1)H^{T}$$

where $\hat{S}(k)$ is the statistical sampling variance of S(k)

(20) $$\hat{S}(k)=\frac{1}{k-1}\sum_{i=1}^{k}\left[M(i)-\bar{M}(i)\right]{\left[M(i)-\bar{M}(i)\right]}^{T}$$

where $\bar{M}(i)$ is statistical sampling mean of innovation.

$$\bar{M}(i)=\frac{1}{i}\sum_{j=1}^{i}\tilde{M}(j)$$

The updated state estimate at time $t_{k}$ is

(21) $$\hat{X}(k|k)=\hat{X}(k|k-1)+K_{k}\left[Z(k)-H_{k}\hat{X}(k|k-1)\right]$$

where $K_{k}$ is the Kalman gain

(22) $$K_{k}=P\left(k|k-1\right)H{\left(k\right)}^{T}{\left[H\left(k\right)P\left(k|k-1\right)H{\left(k\right)}^{T}+R\left(k\right)\right]}^{-1}$$

The updated state covariance at time $t_{k}$ is

(23) $$P\left(k|k\right)=\left[I-K_{k}H\right]P\left(k|k-1\right)$$

By initializing the initial state $\hat{X}\left(0\right)$ and covariance $\hat{P}\left(0\right)$, the state of the underwater target can be estimated through Equations (15)–(23).

## 4. Estimation of AOU

### 4.1. Analysis of AOU Distribution

Let Δx and Δy denote the error of the estimated position along the x and y axis, respectively. The error of the estimated position has a two-dimensional Gaussian distribution [36] and its probability distribution function is

$$f\left(\Delta x,\Delta y\right)=\frac{1}{2\pi \sigma_{X}\sigma_{Y}\sqrt{1-\rho^{2}}}\cdot \exp\left[-\frac{1}{2(1-\rho^{2})}\right]\left[\frac{{\left(\Delta x\right)}^{2}}{\sigma_{X}^{2}}-\frac{2\rho (\Delta x)(\Delta y)}{\sigma_{X}\sigma_{Y}}+\frac{{\left(\Delta y\right)}^{2}}{\sigma_{Y}^{2}}\right]$$

where $\sigma_{X}^{2}$ and $\sigma_{Y}^{2}$ represent the error variance of the location estimate along x and y axis, ρ is the correlation coefficient between Δx and Δy, which is generally non-zero. The distribution of the target position estimates corresponds to AOU of the target, the contour curve of which can be described by elliptic curve with follow equation [36]

$$m^{2}=\frac{{\left(\Delta x\right)}^{2}}{\sigma_{X}^{2}}-\frac{2\rho (\Delta x)(\Delta y)}{\sigma_{X}\sigma_{Y}}+\frac{{\left(\Delta y\right)}^{2}}{\sigma_{Y}^{2}}$$

where m is coefficient of AOU ellipse determined by given confidence probability. As the correlation coefficient ρ is generally non-zero, Δx and Δy are not statistically independent. Therefore, it is necessary to apply quadratic components corresponding to the major and minor axis, respectively, to describe the elliptical AOU.

### 4.2. Algorithm of AOU Estimation

The center of the AOU is the predicted position which can be obtained from the state prediction $\hat{X}(k|k-1)$ of Section 3.4. If the error of the predicted positions along the x and y axis are uncorrelated, then the target position error ellipse has an inclination angle θ of 0 degree. The error covariance matrix has the form

$$P=\left[\begin{array}{cc} s_{1}{}^{2} & 0\\ 0 & s_{2}{}^{2} \end{array}\right]$$

where $s_{1}$ and $s_{2}$ denote the semi-major axis and semi-minor axis of the error ellipse, respectively, and the inclination angle θ is the angle between the major axis and the *x*-axis direction. 

Since the errors of the target position estimation along the x and y axis are generally related in practice, the position error covariance matrix $P^{\prime }$ can be obtained from the state prediction covariance P(k|k−1) in Section 3.4, $P^{\prime }$ has an inclination angle θ. Then, the rotation matrix V, which transforms matrix P into $P^{\prime }$, is [37]

$$V=\left[\begin{array}{cc} \cos\theta & -\sin\theta \\ \sin\theta & \cos\theta \end{array}\right]$$

By solving the rotation matrix V, the inclination angle θ can be obtained. The rotation matrix V is solved according to its physical meaning. By calculating the eigenvalues and corresponding eigenvectors of V, the square of the semi-major axis can be acquired as the maximum eigenvalue, while the square of semi-minor axis is the minimum eigenvalue, and V is the matrix of eigenvector combinations corresponding to the eigenvalues as

$$V=\left[\begin{array}{cc} v_{1} & v_{2} \end{array}\right]=\left[\begin{array}{cc} \cos\theta & -\sin\theta \\ \sin\theta & \cos\theta \end{array}\right]$$

where $v_{1}$ is the eigenvector corresponding to eigenvalue $s_{1}{}^{2}$ and $v_{2}$ is the eigenvector corresponding to eigenvalue $s_{2}{}^{2}$. That is

(24) $$v_{1}={\left[\begin{array}{cc} \cos\theta & \sin\theta \end{array}\right]}^{T},v_{2}={\left[\begin{array}{cc} -\sin\theta & \cos\theta \end{array}\right]}^{T}$$

According to the obtained eigenvectors, the inclination angle of AOU ellipse can be calculated.

As m is the coefficient of the AOU ellipse, the AOU under different coefficients m correspond to different confidence probabilities [38]. Since the error of the estimated target position is normally distributed approximately, the confidence probability corresponding to one times the semi-major axis, the minor semi-axis of AOU ellipse (m=1) is about 39.3%, and the confidence probability is 86.5% when the coefficient m equals to 2 while the confidence probability is 98.9% when m is 3.

The coefficient m of elliptical AOU with confidence probability p is given by [36]

(25) $$m=\sqrt{-2\ln(1-p)}$$

which indicates that when the confidence level is p; the semi-major axis and semi-minor axis of AOU are m times of $s_{1}$ and $s_{2}$.

## 5. Simulation Results

In this section, the results of computer simulation are presented to demonstrate the performance of the proposed algorithm. Scenarios for tracking a moving target are examined. The classical assumption is that favorable environmental conditions facilitate the availability of range and bearing update at each sampling interval. In the simulation, it was assumed that the target was moving with uniform straight-line motion at a constant velocity. The underwater target tracking solution estimated by SMM algorithm was evaluated with the scenarios shown in Table 1. The sampling interval $T_{s}$ for short-range targets was 10 s, and 30 s for long-range targets. Measurement errors were assumed to be zero-mean, Gaussian distributed and uncorrelated. To illustrate the effect of measurement error, four kinds of range and bearing measurements were chosen, i.e., $P_{e1}$: $\sigma_{r}=0.01\cdot r$ and $\sigma_{b}=1^{\circ }$, $P_{e2}$: $\sigma_{r}=0.02\cdot r$ and $\sigma_{b}=1^{\circ }$, $P_{e3}$: $\sigma_{r}=0.01\cdot r$ and $\sigma_{b}=2^{\circ }$, $P_{e4}$: $\sigma_{r}=0.02\cdot r$ and $\sigma_{b}=2^{\circ }$, where $\sigma_{r}$ and $\sigma_{b}$ are the standard deviation of range and bearing measurements respectively, and r is the target range. The simulation compared the tracking performance of the adaptive Kalman filter and the SMM algorithm proposed in this paper.

### 5.1. Simulation of Target Tracking

**Figure 2:**
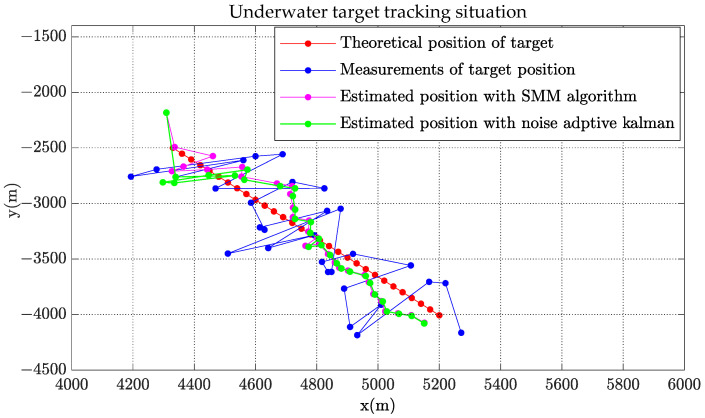
Target and observer position with estimates.

**Figure 3:**
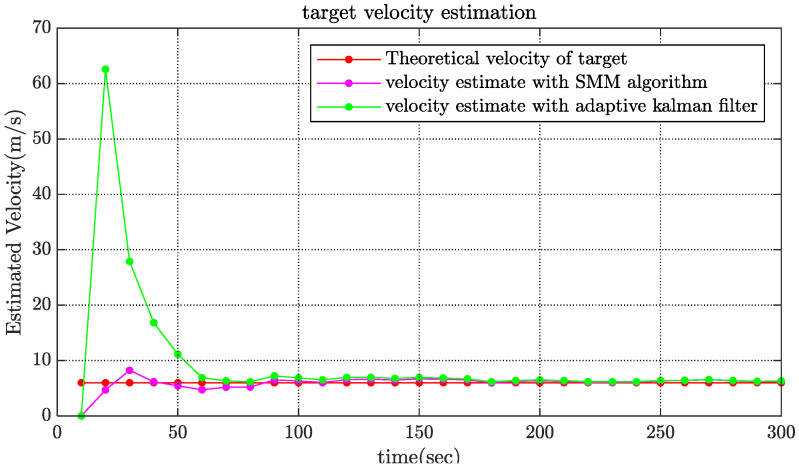
Comparison of estimated velocity.

**Figure 4:**
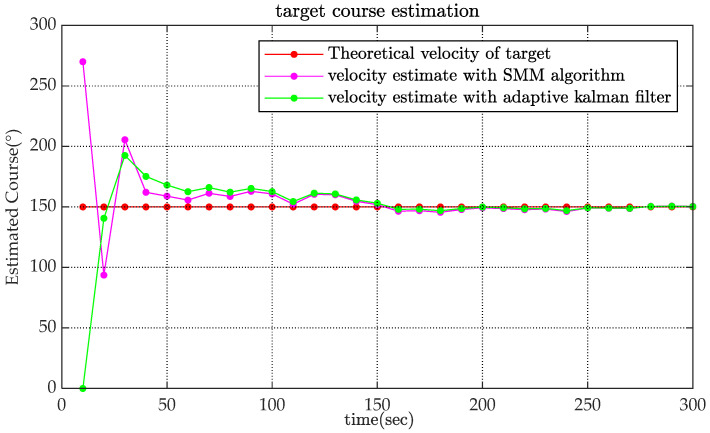
Comparison of estimated course.

Figure 2, Figure 3 and Figure 4 show the state estimation results of the underwater target in a single simulation with scenario 1. Figure 2 shows the target trajectory tracking diagram, and the estimation results of target velocity and course are shown in Figure 3 and Figure 4. It can be seen that the algorithm in this paper can effectively estimate the state of the target and has better performance than adaptive Kalman filter.

**Figure 5:**
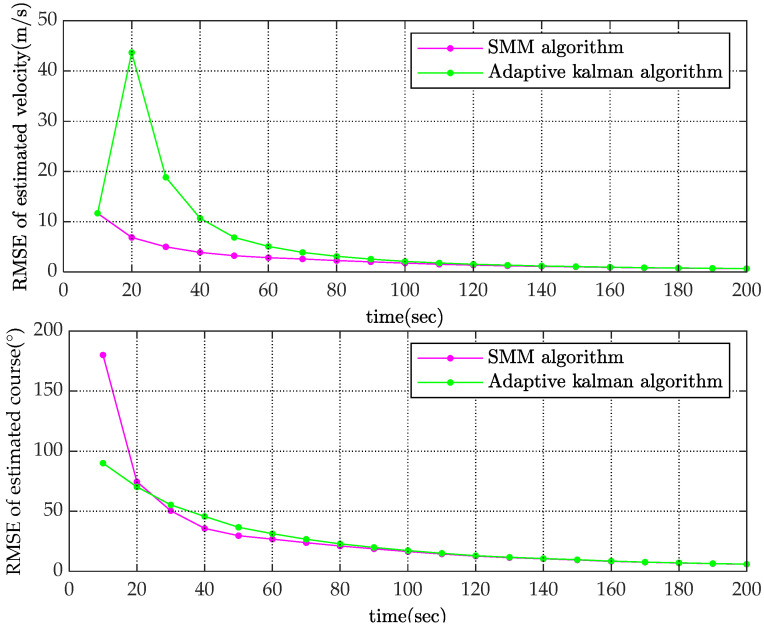
Comparison of RMSE for estimated velocity and course.

**Figure 6:**
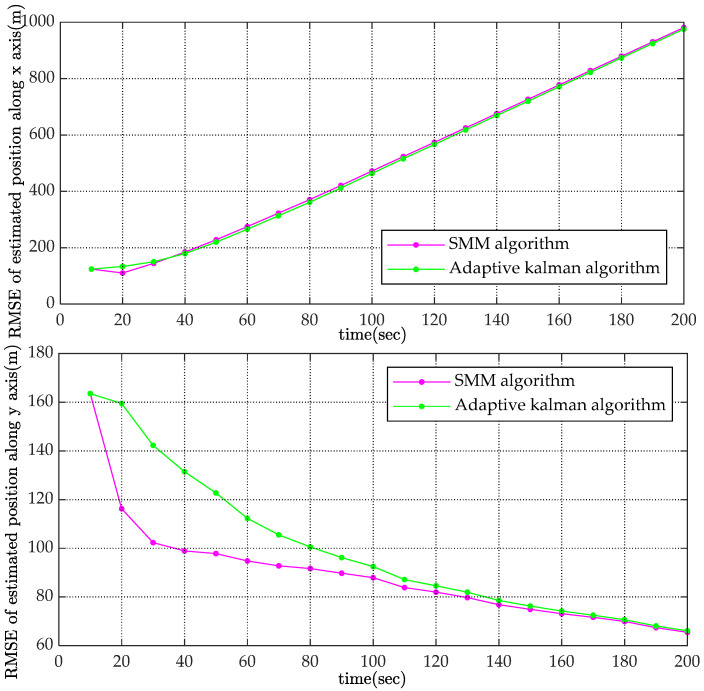
Comparison of RMSE for estimated position along x axis and y axis.

To reduce the effect of random error, the simulation was carried out M(M=10,000) times. With root mean square error as the statistic to indicate performance, Monte-Carlo results are shown in Figure 5 and Figure 6.

The RMSE (root mean square error) of the i−th observation was calculated as follows [38]:

$$RMSE_{i}=\frac{1}{M}\sqrt{\sum_{k=1}^{M}{\left(x_{i}-{\tilde{x}}_{i}{}^{k}\right)}^{2}}$$

where $x_{i}$ is the true value and ${\tilde{x}}_{i}{}^{k}$ denotes the observed value.

The RMSE values of the estimated velocity and course are shown in Figure 5. Compared with the adaptive Kalman filter, the SMM tracking algorithm could reduce the estimated velocity error by more than 50% and reduce the estimated course error by 20% at the initial stage of tracking. The estimated velocity RMSE of x-coordinate and y-coordinate are shown in Figure 6. It is obviously that the estimated position of SMM tracking algorithm has a better performance, especially on velocity estimation.

**Figure 7:**
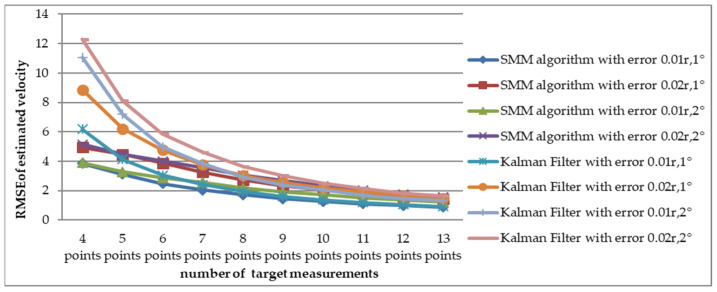
Comparison of estimated velocity RMSE between SMM algorithm and adaptive Kalman filter versus different detection error (Scenario 1).

**Figure 8:**
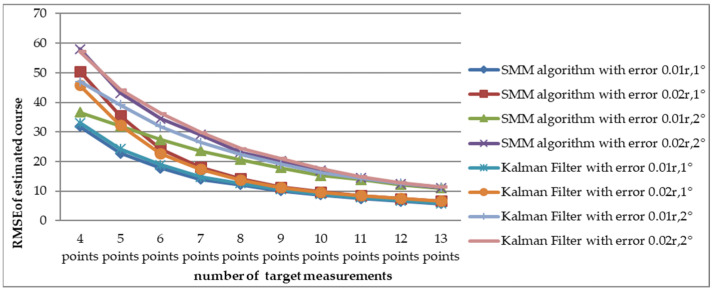
Comparison of estimated course RMSE between SMM algorithm and adaptive Kalman filter versus different detection error (Scenario 1).

**Figure 9:**
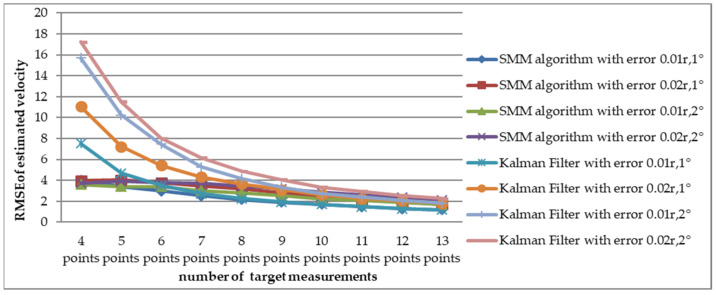
Comparison of estimated velocity RMSE between SMM algorithm and adaptive Kalman filter versus different detection error (Scenario 7).

**Figure 10:**
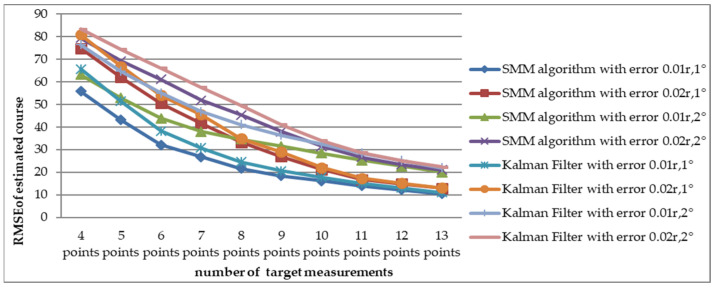
Comparison of estimated course RMSE between SMM algorithm and adaptive Kalman filter versus different detection error (Scenario 7).

To compare the RMSE of estimated velocity and course with the SMM algorithm and adaptive Kalman filter, a Monte Carlo simulation was carried out 10,000 times for short-range target tracking (Scenario 1) and long-range target tracking (Scenario 7). The RMSE of estimated velocity for the short-range target with different detection errors is shown in Figure 7. In the considered scenario, the RMSE of velocity decreased as the number of measurements increased. The change of estimated velocity with the SMM algorithm was not as dramatic as with the Kalman filter at the beginning of tracking and has a smaller RMSE from four to eight points. For example, when the amount of target detection was 4 and the detection error was $P_{e4}$($\sigma_{r}=0.02\cdot r$, $\sigma_{b}=2^{\circ }$), RMSE with the SMM algorithm was 5.1632 m/s and the result with Kalman filter was 12.29 m/s. The RMSE comparison of estimated course is shown in Figure 8, which illustrates that the RMSE of course decreased as the number of measurements increased. As is shown in Figure 9, RMSE of estimated velocity for the long-range target SMM algorithm was 3.7324 m/s, while the result was 17.2346 m/s with the Kalman filter, which means velocity RMSE can be reduced more than 50% at the beginning of tracking, which is significant for time-sensitive target tracking. The RMSE of estimated course for a long-range target with different detection errors is also shown in Figure 10. Similarly, the RMSE of the course decreased as the number of measurements increased. For the short-range target, the performance of the estimated course with the SMM algorithm was a little better than that with the Kalman filter. On the other hand, for the long-range target, RMSE of estimated course with the SMM algorithm was reduced by more than 15% compared with the Kalman filter for the initial stage of tracking. 

### 5.2. Simulation of AOU Estimation

**Figure 11:**
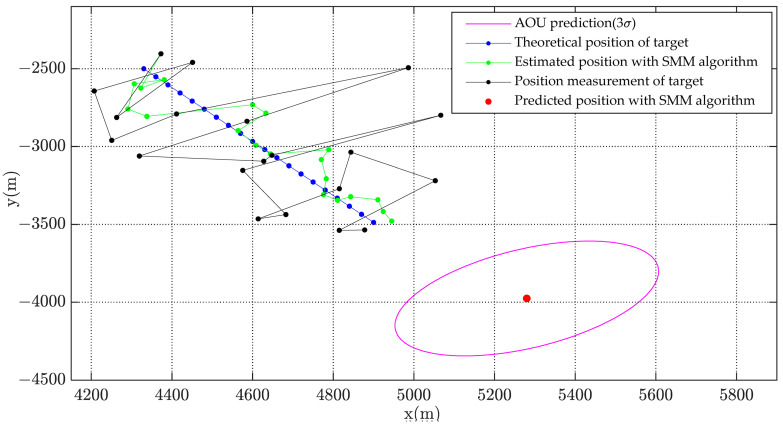
Underwater target tracking and its AOU.

In order to show the effectiveness of AOU estimation algorithm, the simulation is carried out. The simulation condition is the same to the state estimation simulation with scenarios in Table 1. The movement situation of Scenario 1 with the detection error $P_{e4}$ is shown in Figure 11 and the AOU of underwater target is predicted after 200 s movement.

**Figure 12:**
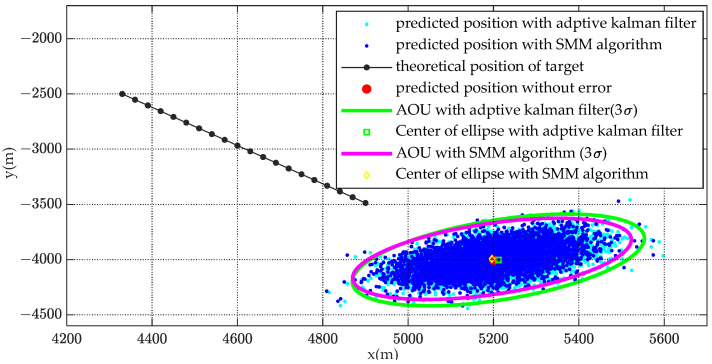
AOU coverage with 20,000 simulated random points.

**Figure 13:**
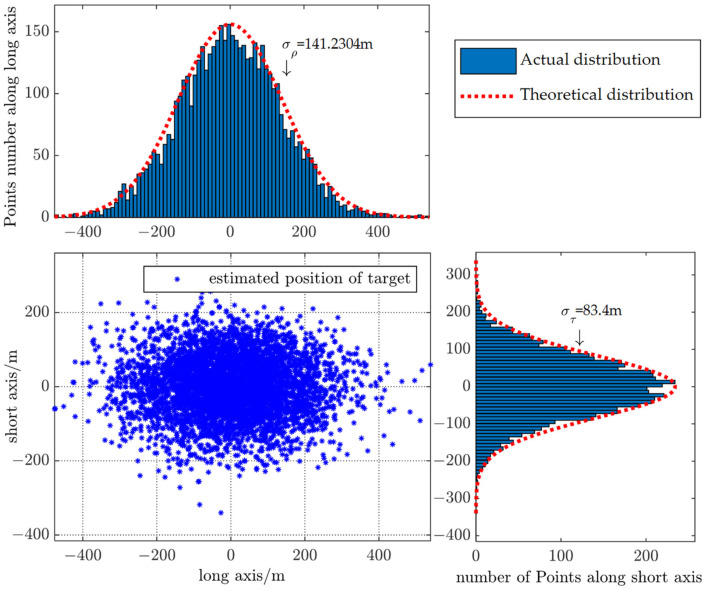
Marginal distribution of AOU.

In order to analyze the distribution of predicted target position and reduce the influence of random error, the Monte Carlo simulation was carried out 10,000 times. The simulation results of AOU estimation are shown in Figure 12 and Figure 13.

Figure 12 shows the distribution of estimated target position with Monte Carlo simulation. The magenta ellipse is the contour of AOU (under confidence probability 98.9%) with SMM algorithm while the green ellipse is the contour of AOU with adaptive Kalman filter. It is obviously that the contour of calculated AOU ellipse agrees well with the distribution of estimated target position. The estimated AOU with SMM algorithm is more centralized than that with adaptive Kalman filter. 

Figure 13 illustrates the marginal distribution of AOU along major and minor axis with bar graph. The red curve is the marginal probability density curve satisfying normal distribution generated by the long and short axes of the target AOU calculated with the method in this paper. As is shown in Figure 13, the actual distribution of predicted target position is consistent with the assumption of two-dimensional normal distribution, which verifies the effectiveness of the algorithm.

In order to analyze the performance of AOU estimation with different affecting factors, the simulation study involves the evaluation of 12 scenarios of short-range and long-range target for various values of $P_{e}$. The comparison between RMSE of predicted position, semi-major of AOU, and semi-major of AOU after 200 s is shown in Table 2 with different values of $P_{e}$ for short-range target Scenario 1 to Scenario 6 of Table 1. The results for long-range target is shown in Table 3.

**Figure 14:**
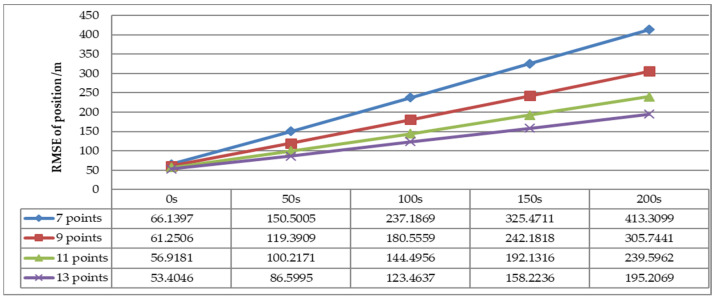
RMSE of predicted position versus different prediction time (Scenario 1, $P_{e}$ = $P_{e1}$).

**Figure 15:**
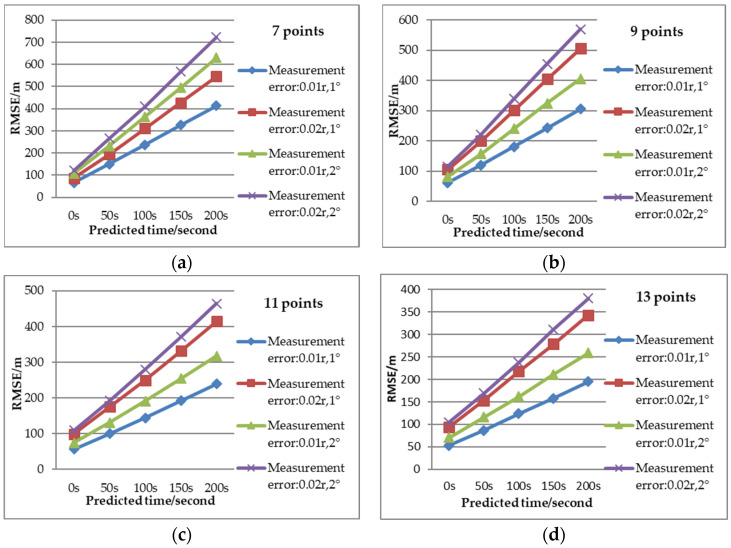
RMSE comparison of predicted position versus different detection error. (**a**) RMSE of predicted position with seven points. (**b**) RMSE of predicted position with nine points. (**c**) RMSE of predicted position with eleven points. (**d**) RMSE of predicted position with thirteen points.

The influence of prediction time and number of measurements on the RMSE of the predicted position is analyzed in Figure 14 with Scenario 1 (for detection error $P_{e1}$). As is shown, the RMSE of predicted position increased longer prediction time. With the increase of the number of measurements, the prediction error became smaller. As is shown in Figure 15, the RMSE of the predicted position for different numbers of measurements (7, 9, 11 and 13 points) varies with detection errors as listed in Figure 15. It is apparent that the predicted position is more accurate with lower RMSE when the detection error is smaller. 

**Figure 16:**
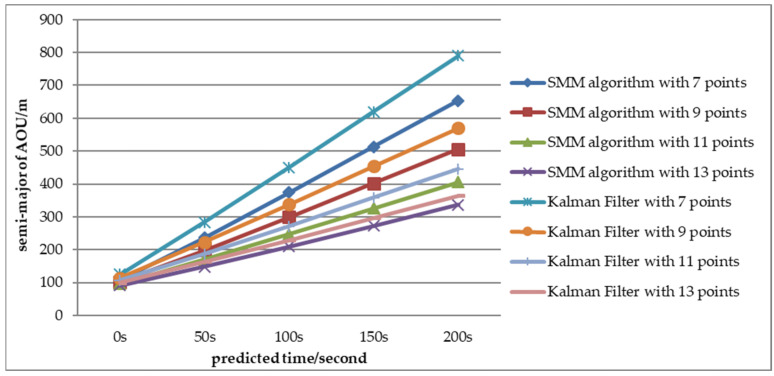
AOU semi-major comparison between the SMM algorithm and the adaptive Kalman filter ($P_{e}$ = $P_{e4}$).

**Figure 17:**
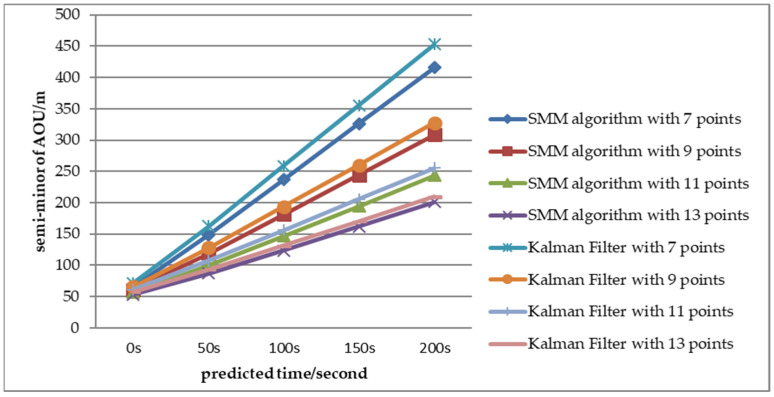
AOU semi-minor comparison between the SMM algorithm and the adaptive Kalman filter ($P_{e}$ = $P_{e4}$).

**Figure 18:**
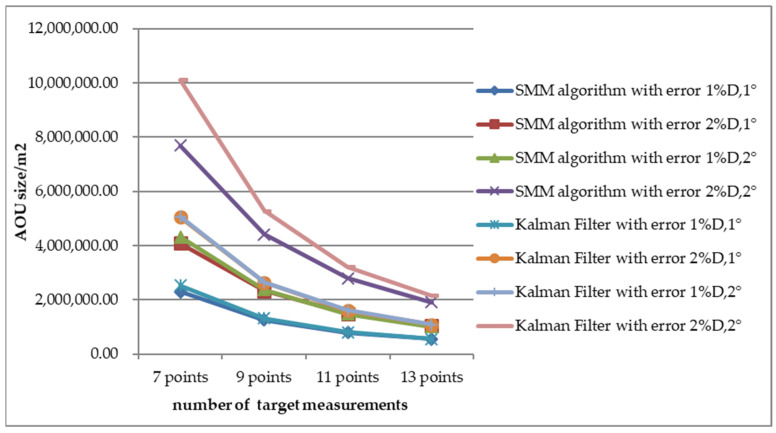
Comparison of estimated AOU size with different measurement errors.

To evaluate the performance of AOU estimation, the AOU comparison between SMM algorithm and adaptive Kalman Filter with CV motion is shown in Figure 16, Figure 17 and Figure 18 with Scenario 1. The semi-major and semi-minor of AOU with different number of measurements (7, 9, 11, and 13 points) are shown Figure 16 and Figure 17. The semi-major and semi-minor of AOU with the SMM algorithm were smaller than with the adaptive Kalman filter. With fewer measurements, the SMM algorithm was more significant than with the adaptive Kalman Filter, especially with larger measurement error. For example, when the prediction time was 200 s and the number of measurements was seven, the semi-major of AOU with SMM algorithm was 653.8 m while the result with the Kalman Filter was 789.9 m, which indicates that the SMM algorithm can shrink the semi-major of AOU by more than 20.8%. Similar results of semi-minor can be obtained from Figure 17. From Figure 17, it can be seen that under different error conditions, the estimated AOU of the SMM algorithm was more concentrated than that of the Kalman Filter. When the prediction time is longer, the AOU is larger and SMM algorithm performs better than the Kalman Filter. A comparison of AOU with prediction time 200 s between SMM algorithm and Kalman filter is shown in Figure 18. When the measurement error is large ($P_{e4}$), SMM algorithm apparently performs better than Kalman filter, especially at the initial stage of tracking, where the size of AOU for the SMM algorithm is reduced 25% with seven points.

## 6. Conclusions

In this paper, we propose an algorithm of AOU estimation for an underwater moving target based on a stochastic maneuvering motion model. The stochastic maneuvering motion model was established based on the Langevin equation, and a stochastic differential equation was applied to describe the underwater target motion. By applying the stochastic maneuvering motion model to noise adaptive Kalman filter, the underwater target state was estimated. On the basis of the physical significance of error covariance matrix from state estimation, the equation of AOU estimation was deduced and AOU of the underwater moving target under different confidence levels could be estimated. The target state estimation accuracy was studied in terms of RMSE, and AOU estimation was evaluated in terms of predicted position and error eclipse. From the simulation results of tracking, it can be deduced that the SMM algorithm outperformed the adaptive Kalman filter applying CV motion. As the RMSE of estimated velocity with SMM algorithm can be reduced more than 50% at the beginning of tracking, this provides a gentle change in velocity estimation at the initial stage of tracking under a large detection error, which helps to reduce the error of the predicted position and leads to a more concentrated AOU distribution. Monte Carlo simulation of AOU estimation verified the effectiveness of the algorithm in that the predicted target position was consistent with the assumption of a two-dimensional normal distribution and could be defined by the confidence level. Furthermore, the size of AOU with SMM algorithm was reduced by 25% compared to the adaptive Kalman Filter during the initial tracking stage with a larger measurement error, which is very significant for time-sensitive target tracking and attack. The proposed algorithm can provide predicted position and AOU of an underwater target for weapon strike and combat decision-making.

In fact, the proposed algorithm can estimate the track of a maneuvering underwater target by changing the model parameters. The combination of maneuver characteristics and model parameters of a maneuvering target is within the scope of future work.

## Figures and Tables

**Table 1:** Scenarios of underwater target at close range used for performance evaluation.

Scenario	Initial Range (m)	Initial Bearing (deg)	Target Speed (m/s)	Target Course (deg)	Observer Speed (m/s)	Observer Course (deg)	Sampling Interval (s)
1	5000	120	6	150	5	150	10
2	5000	120	6	160	5	150	10
3	5000	120	8	150	5	150	10
4	5000	150	8	120	5	150	10
5	5000	90	8	160	5	150	10
6	5000	120	4	210	5	150	10
7	20,000	60	4	270	5	150	30
8	20,000	90	4	310	5	150	30
9	20,000	90	8	310	5	150	30
10	20,000	60	6	330	5	150	30
11	20,000	60	8	150	5	150	30
12	20,000	120	6	150	5	150	30

**Table 2:** RMSE of estimated position and AOU with different scenarios versus detection error values (close-range underwater target).

$P_{e}$	N	Scenario	1	2	3	4	5	6
$\sigma_{r}=0.01\cdot r$ $\sigma_{b}=1^{\circ }$	7	$\sigma_{Dp}$	413.310	417.128	426.485	426.113	435.141	399.983
		$s_{1}$	368.170	366.807	371.960	370.608	366.821	356.518
		$s_{2}$	220.939	220.120	223.271	222.543	220.192	213.861
	9	$\sigma_{Dp}$	305.744	306.727	316.616	314.058	307.680	293.429
		$s_{1}$	271.807	270.492	275.775	274.383	270.475	259.865
		$s_{2}$	163.175	162.431	165.482	164.794	162.495	156.329
	11	$\sigma_{Dp}$	239.596	241.977	246.063	243.821	240.470	224.455
		$s_{1}$	214.403	213.054	218.335	216.966	213.075	202.480
		$s_{2}$	130.486	129.753	132.693	132.063	129.849	123.910
$\sigma_{r}=0.01\cdot r$ $\sigma_{b}=2^{\circ }$	7	$\sigma_{Dp}$	629.941	643.475	652.640	647.520	765.587	630.345
		$s_{1}$	652.417	650.370	657.978	655.598	650.127	635.025
		$s_{2}$	221.181	220.399	223.555	222.985	220.547	214.126
	9	$\sigma_{Dp}$	505.424	504.768	522.597	518.075	553.676	491.760
		$s_{1}$	504.938	502.536	511.684	508.685	502.245	484.083
		$s_{2}$	163.336	162.593	165.674	165.202	162.817	156.505
	11	$\sigma_{Dp}$	414.391	411.537	417.133	419.053	440.183	395.159
		$s_{1}$	404.723	402.151	411.919	408.788	401.771	382.323
		$s_{2}$	130.582	129.881	132.822	132.445	130.144	124.057
$\sigma_{r}=0.02\cdot r$ $\sigma_{b}=1^{\circ }$	7	$\sigma_{Dp}$	546.073	539.106	564.347	563.965	547.174	514.209
		$s_{1}$	415.476	414.109	419.742	418.352	414.146	402.624
		$s_{2}$	368.096	366.867	371.954	370.730	366.919	356.511
	9	$\sigma_{Dp}$	405.472	401.254	415.828	412.366	404.800	380.204
		$s_{1}$	308.233	306.777	312.739	311.213	306.809	294.716
		$s_{2}$	271.811	270.528	275.804	274.494	270.582	259.869
	11	$\sigma_{Dp}$	317.670	316.319	323.708	321.294	315.395	289.176
		$s_{1}$	243.213	241.650	247.655	246.225	241.742	229.578
		$s_{2}$	214.418	213.055	218.316	217.109	213.166	202.498
$\sigma_{r}=0.02\cdot r$ $\sigma_{b}=2^{\circ }$	7	$\sigma_{Dp}$	723.203	722.177	749.495	757.848	839.797	709.008
		$s_{1}$	653.884	651.785	659.553	657.448	651.929	636.578
		$s_{2}$	415.719	414.157	419.994	418.586	414.381	402.860
	9	$\sigma_{Dp}$	568.903	570.687	592.456	580.603	612.238	546.415
		$s_{1}$	506.557	504.110	513.323	510.839	504.206	485.674
		$s_{2}$	308.430	306.856	312.899	311.507	307.057	294.884
	11	$\sigma_{Dp}$	463.964	459.507	467.443	472.610	483.452	435.332
		$s_{1}$	406.165	403.642	413.466	410.863	403.629	383.800
		$s_{2}$	243.307	241.796	247.821	246.488	241.960	229.719

Note: N, number of measured points; $\sigma_{Dp}$, RMSE of predicted position; $s_{1}$, semi-major axis of AOU; $s_{2}$, semi-minor axis of AOU.

**Table 3:** RMSE of estimated position and AOU with different scenarios versus detection error values (long-range underwater target).

$P_{e}$	N	Scenario	7	8	9	10	11	12
$P_{e1}$: $\sigma_{r}=0.01\cdot r$ $\sigma_{b}=1^{\circ }$	7	$\sigma_{Dp}$	413.310	417.128	426.485	426.113	435.141	399.983
		$s_{1}$	368.170	366.807	371.960	370.608	366.821	356.518
		$s_{2}$	220.939	220.120	223.271	222.543	220.192	213.861
	9	$\sigma_{Dp}$	305.744	306.727	316.616	314.058	307.680	293.429
		$s_{1}$	271.807	270.492	275.775	274.383	270.475	259.865
		$s_{2}$	163.175	162.431	165.482	164.794	162.495	156.329
	11	$\sigma_{Dp}$	239.596	241.977	246.063	243.821	240.470	224.455
		$s_{1}$	214.403	213.054	218.335	216.966	213.075	202.480
		$s_{2}$	130.486	129.753	132.693	132.063	129.849	123.910
$P_{e2}$: $\sigma_{r}=0.01\cdot r$ $\sigma_{b}=2^{\circ }$	7	$\sigma_{Dp}$	629.941	643.475	652.640	647.520	765.587	630.345
		$s_{1}$	652.417	650.370	657.978	655.598	650.127	635.025
		$s_{2}$	221.181	220.399	223.555	222.985	220.547	214.126
	9	$\sigma_{Dp}$	505.424	504.768	522.597	518.075	553.676	491.760
		$s_{1}$	504.938	502.536	511.684	508.685	502.245	484.083
		$s_{2}$	163.336	162.593	165.674	165.202	162.817	156.505
	11	$\sigma_{Dp}$	414.391	411.537	417.133	419.053	440.183	395.159
		$s_{1}$	404.723	402.151	411.919	408.788	401.771	382.323
		$s_{2}$	130.582	129.881	132.822	132.445	130.144	124.057
$P_{e3}$: $\sigma_{r}=0.02\cdot r$ $\sigma_{b}=1^{\circ }$	7	$\sigma_{Dp}$	546.073	539.106	564.347	563.965	547.174	514.209
		$s_{1}$	415.476	414.109	419.742	418.352	414.146	402.624
		$s_{2}$	368.096	366.867	371.954	370.730	366.919	356.511
	9	$\sigma_{Dp}$	405.472	401.254	415.828	412.366	404.800	380.204
		$s_{1}$	308.233	306.777	312.739	311.213	306.809	294.716
		$s_{2}$	271.811	270.528	275.804	274.494	270.582	259.869
	11	$\sigma_{Dp}$	317.670	316.319	323.708	321.294	315.395	289.176
		$s_{1}$	243.213	241.650	247.655	246.225	241.742	229.578
		$s_{2}$	214.418	213.055	218.316	217.109	213.166	202.498
$P_{e4}$: $\sigma_{r}=0.02\cdot r$ $\sigma_{b}=2^{\circ }$	7	$\sigma_{Dp}$	723.203	722.177	749.495	757.848	839.797	709.008
		$s_{1}$	653.884	651.785	659.553	657.448	651.929	636.578
		$s_{2}$	415.719	414.157	419.994	418.586	414.381	402.860
	9	$\sigma_{Dp}$	568.903	570.687	592.456	580.603	612.238	546.415
		$s_{1}$	506.557	504.110	513.323	510.839	504.206	485.674
		$s_{2}$	308.430	306.856	312.899	311.507	307.057	294.884
	11	$\sigma_{Dp}$	463.964	459.507	467.443	472.610	483.452	435.332
		$s_{1}$	727.026	712.529	692.990	752.298	748.716	754.345
		$s_{2}$	438.710	429.487	418.135	456.018	451.763	455.219

Note: N, number of measured points; $\sigma_{Dp}$, RMSE of predicted position; $s_{1}$, semi-major axis of AOU; $s_{2}$, semi-minor axis of AOU.
